# Impact of guideline changes on adoption of hypofractionation and breast cancer patient characteristics in the randomized controlled HYPOSIB trial

**DOI:** 10.1007/s00066-020-01730-9

**Published:** 2020-12-15

**Authors:** David Krug, Reinhard Vonthein, Andreas Schreiber, Alexander D. Boicev, Jörg Zimmer, Reinhold Laubach, Nicola Weidner, Stefan Dinges, Matthias Hipp, Ralf Schneider, Evelyn Weinstrauch, Thomas Martin, Juliane Hörner-Rieber, Denise Olbrich, Alicia Illen, Nicole Heßler, Inke R. König, Kathrin Dellas, Jürgen Dunst

**Affiliations:** 1 Klinik für Strahlentherapie, Universitätsklinikum Schleswig-Holstein Campus Kiel, Arnold-Heller-Str. 3, 24105 Kiel, Germany; 2 Institut für Medizinische Biometrie und Statistik, Universität zu Lübeck, Universitätsklinikum Schleswig-Holstein, Lübeck, Germany; 3 Praxis für Strahlentherapie Dr. med. Andreas Schreiber, Dresden, Germany; 4 Klinik für Strahlentherapie und Radioonkologie, Heinrich-Braun-Klinikum Zwickau, Zwickau, Germany; 5 Klinik für Radio-Onkologie, St. Marien-Krankenhaus Siegen, Siegen, Germany; 6 Klinik für Strahlentherapie, Universitätsklinikum Tübingen, Tübingen, Germany; 7 Klinik für Strahlentherapie & Radioonkologie, Klinikum Lüneburg, Lüneburg, Germany; 8 Klinik für Strahlentherapie, Klinikum Amberg, Amberg, Germany; 9 Klinik für Strahlentherapie, Helios-Kliniken Schwerin, Schwerin, Germany; 10 Praxis für Radioonkologie, Johanniter-Zentren für Medizinische Versorgung Stendal, Stendal, Germany; 11 Medizinisches Versorgungszentrum Fachbereich RadioOnkologie, Klinikum Bremen-Mitte, Bremen, Germany; 12 RadioOnkologie und Strahlentherapie, UniversitätsKlinikum Heidelberg, Heidelberg, Germany; 13 ZKS Lübeck (Zentrum für klinische Studien Lübeck), Universität zu Lübeck, Lübeck, Germany

**Keywords:** Guideline implementation, Breast cancer, Radiotherapy, Hypofractionation

## Abstract

**Purpose:**

Hypofractionated radiotherapy is the standard of care for adjuvant whole breast radiotherapy (RT). However, adoption has been slow. The indication for regional nodal irradiation has been expanded to include patients with 0–3 involved lymph nodes. We investigated the impact of the publication of the updated German S3 guidelines in 2017 on adoption of hypofractionation and enrollment of patients with lymph node involvement within a randomized controlled phase III trial.

**Methods:**

In the experimental arm of the HYPOSIB trial (NCT02474641), hypofractionated RT with simultaneous integrated boost (SIB) was used. In the standard arm, RT could be given as hypofractionated RT with sequential boost (HF_seq_), normofractionated RT with sequential boost (NF_seq_), or normofractionated RT with SIB (NF_SIB_). The cutoff date for the updated German S3 guidelines was December 17, 2017. Temporal trends were analyzed by generalized linear regression models. Multiple logistic regression models were used to investigate the influence of time (prior to/after guideline) and setting (university hospital/other institutions) on the fractionation patterns.

**Results:**

Enrollment of patients with involved lymph nodes was low throughout the trial. Adoption of HF_seq_ increased over time and when using the guideline publication date as cutoff. Results of the multiple logistic regressions showed an interaction between time and setting. Furthermore, the use of HF_seq_ was significantly more common in university hospitals.

**Conclusion:**

The use of HF_seq_ in the standard arm increased over the course of the HYPOSIB trial and after publication of the S3 guideline update. This was primarily driven by patients treated in university hospitals. Enrolment of patients with lymph node involvement was low throughout the trial.

## Introduction

For over more than a quarter of a century, conventional fractionation with a total dose of 50 Gy in 25 to 28 fractions over 5 to 6 weeks was the standard of care for adjuvant whole-breast radiotherapy after breast-conserving surgery. About 10 to 15 years ago, however, several large randomized controlled trials from Britain and Canada demonstrated that moderate hypofractionation (e.g., 40 to 42.5 Gy in 15 to 16 fractions) with moderate acceleration (reducing overall treatment time to 3 weeks) is an alternative fractionation regimen with equal efficacy and late toxicity but slightly better acute tolerance [1–4]. The radiobiological basis for these results is a hypothesized low α/β value for breast cancer in the range of 3–4 Gy, which was later confirmed by the START trials [5]. Mature results of the Canadian and the British START trials with follow-up of 10 years were published in 2010 and 2013, respectively [5, 6]. Since then, hypofractionation has been gradually introduced into clinical routine and has been recommended in national guidelines. In the German S3 guideline on diagnosis and treatment of breast cancer, hypofractionation was considered as an alternative to conventional fractionation for adjuvant whole-breast radiotherapy in elderly patients with low-risk breast cancer from 2012 [7]. In these guidelines, regional nodal irradiation (RNI) was recommended for patients with four or more involved lymph nodes [7]. Only a minority of patients enrolled into the available phase III trials of hypofractionated adjuvant radiotherapy [8] received regional nodal irradiation. In 2015, two randomized controlled trials showed an improvement of disease-free survival in patients with 0–3 involved lymph nodes by RNI including the internal mammary lymph nodes [9, 10].

In December 2017, the updated German S3 guideline for management of breast cancer was published [11]. Hypofractionated whole-breast radiotherapy was recommended as the standard of care for patients undergoing breast irradiation without RNI [12]. Furthermore, conventionally fractionated RNI including the internal mammary lymph nodes was recommended for patients with 1–3 involved axillary lymph nodes depending on the presence of additional risk factors such as medial/central tumor location, premenopausal status, and negative hormone receptor status.

HYPOSIB (ARO 2013-05, NCT02474641) is a large multicentric randomized non-inferiority trial comparing hypofractionated whole-breast radiotherapy with a simultaneous integrated boost to standard adjuvant whole-breast radiotherapy plus boost. The S3 guideline update became effective during the enrollment period of the HYPOSIB trial after about 60% of the patients had been recruited. Therefore, we sought to investigate whether the guideline changes had an impact on patients’ characteristics and on the choice of fractionation in patients who were randomized to the standard arm.

## Methods

The HYPOSIB trial is a prospective randomized controlled phase III non-inferiority trial of hypofractionated whole-breast radiotherapy with a simultaneous integrated boost (NCT02474641). Patients were eligible if they had histologically proven unilateral unifocal invasive breast cancer treated with guideline-conforming breast-conserving surgery with an indication for adjuvant whole-breast radiotherapy and a tumor bed boost. The tumor bed had to be identifiable in the radiotherapy planning CT. The use of clips demarcating the lumpectomy cavity was not mandatory. Patients had to be ≥18 years and had to have an Eastern Cooperative Oncology Group (ECOG) performance status of ≤2. Exclusion criteria included bilateral breast cancer, extensive seroma, indication for RNI, participation in another clinical trial of radiotherapy and/or experimental drugs ≤4 weeks before enrollment, uncontrolled severe comorbidities with relevance for study participation, and prior malignancies with the exception of successfully treated basal cell carcinoma of the skin and carcinoma in situ of the cervix. Patients were randomized 1:1 to the standard or to the experimental arm. Patients were recruited at 87 radiotherapy departments from Germany and one from Austria.

In the experimental arm, patients received hypofractionated whole-breast radiotherapy with 40 Gy in 16 fractions of 2.5 Gy with an additional simultaneous integrated boost of 0.5 Gy to the tumor bed, resulting in a total dose of 48 Gy in 16 fractions of 3 Gy to the tumor bed (HF_SIB_). This regimen was studied in two prior single-arm phase II trials [13, 14]. In the standard arm, three different fractionation regimens were allowed per choice of the treating physician: normofractionated radiotherapy with a sequential boost (NF_seq_; 50.4 Gy in 28 fractions to the whole breast +10–16 Gy in 5–8 fractions to the tumor bed), normofractionated radiotherapy with a simultaneous integrated boost (NF_SIB_; 50.4 Gy/58.8 Gy or 50.4/63 Gy in 28 fractions), and hypofractionated radiotherapy with a sequential boost (HF_seq_; 42.5 Gy in 16 fractions to the whole breast +10–16 Gy in 5–8 fractions to the tumor bed). The choice of systemic therapy, i.e., chemotherapy, endocrine therapy, or targeted therapy, was at the discretion of the treating physician. Enrollment of patients with involved lymph nodes was allowed; however, RNI was not permitted.

**Fig. 1:**
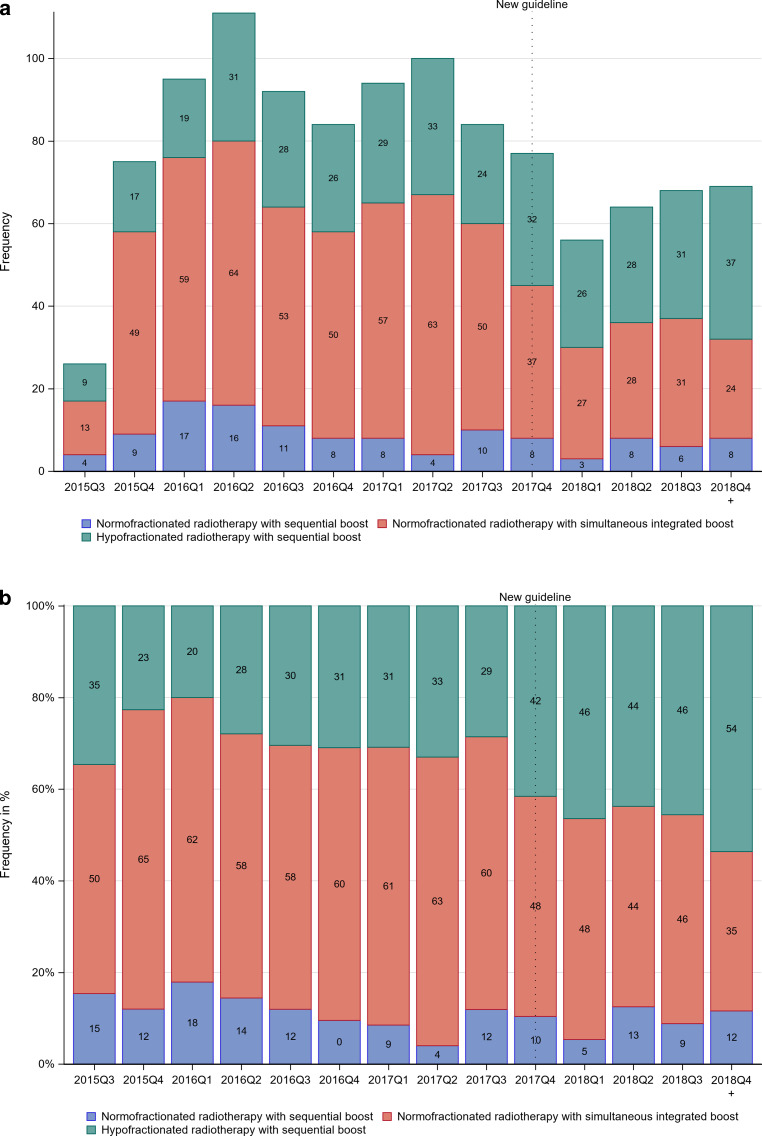
Distribution of fractionation regimens in the standard arm by quarter year (*Q*) of enrollment before and after the S3 guideline update (*dotted line*) in **a** absolute numbers and **b** relative frequencies. The first quarter contains just 5 weeks of recruitment, the last 14 weeks

**Fig. 2:**
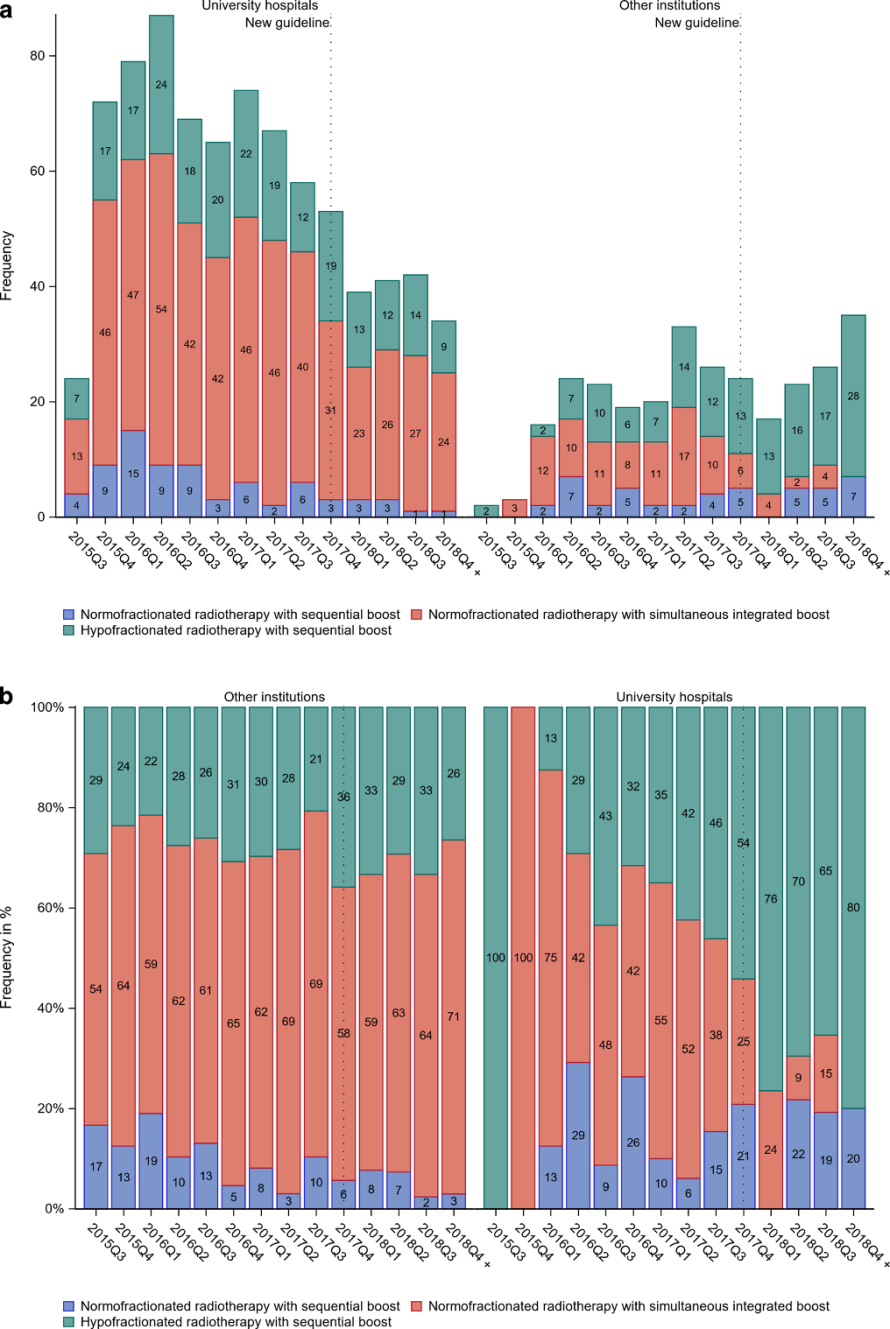
Distribution of fractionation regimens in the standard arm by quarter year (*Q*) of enrollment before and after the S3 guideline update (*dotted line*) and by setting (university hospital vs. other institutions) in **a** absolute numbers and **b** relative frequencies. The first quarter contains just 5 weeks of recruitment, the last 14 weeks

**Fig. 3:**
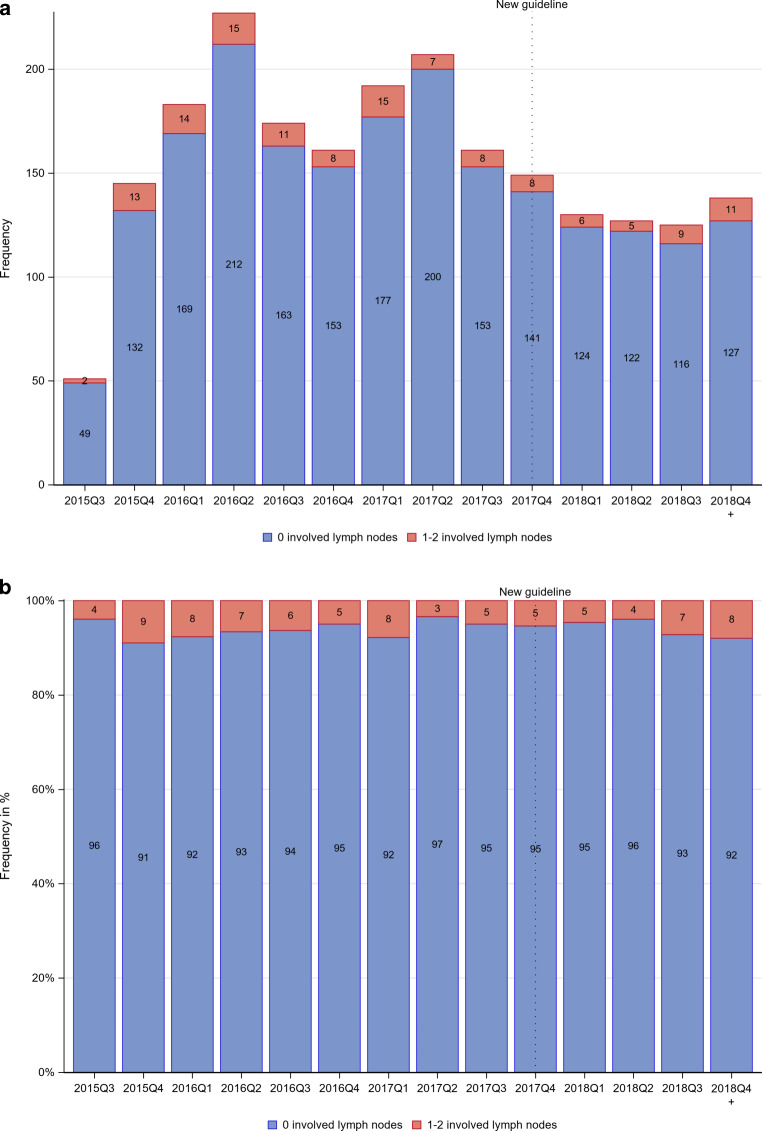
Distribution of nodal involvement by quarter year of enrollment before and after guideline update (*dotted line*) in **a** absolute numbers and **b** relative frequencies in the as treated population. The first quarter contains just 5 weeks of recruitment, the last 14 weeks

The primary endpoint of the HYPOSIB trial is disease-free survival using a non-inferiority design. Secondary endpoints include time to local recurrence, overall survival, acute and chronic toxicity, quality of life, and cosmesis. Here, we conducted a retrospective analysis after the end of accrual to investigate the impact of the updated S3 guideline on the enrollment of patients with involved lymph nodes into the HYPOSIB trial and on fractionation patterns in the standard arm. For simple counts before and after publication of the S3 guideline update, December 19, 2017 was used as the cutoff date [11]. For visualization, lymph node involvement and fractionation patterns are shown as absolute numbers (Fig. 1a, 2a and 3a) and proportions per quarter year of enrollment (Fig. 1b, 2b and 3b). Temporal trends in the enrollment of patients according to nodal involvement and in the use of fractionation patterns in the standard arm were analyzed by generalized linear regression models. Relative risks (RR) and 95% confidence intervals (CI) were calculated. Multiple logistic regression models were used to investigate the influence of time (prior to/after S3 guideline) and setting (university hospital/other institutions) as main effects and the interaction between them on the fractionation pattern adjusted for tumor size (in centimeters), age (in years), and chemotherapy (yes/no). As a dependent variable one treatment regimen of interest (coded 1) is compared to both others (coded 0), resulting in three pairwise comparisons. Effect estimates, standard errors, and *p*-values are reported. The interaction between time and setting is tested for significance with adjustment for multiple testing of the *p*-values according to Bonferroni–Holm. Other *p*‑values are reported for descriptive purposes. Baseline characteristics are listed as absolute numbers and proportions. The analysis set was the as-treated population with the condition that only patients with available treatment information assessed in the radiation plan and at the final examination were considered. A *p*-value <0.05 was considered statistically significant.

## Results

From June 2015 to January 2019, 2323 patients were enrolled in the HYPOSIB trial and randomized to HF_SIB_ (*n* = 1155) or standard of care (*n* = 1168). The as-treated population consists of 2182 patients. Of these, 1095 patients were treated in the standard arm and received NF_seq_, NF_SIB_, or HF_seq_. Three patients in the experimental arm received treatment other than HF_SIB_, and 15 patients in the control arm received HF_SIB_. For 141 patients, data were incomplete at the time of this analysis. For the analysis regarding lymph node status, 2170 patients with known lymph node status were analyzed. Baseline characteristics are shown in Table 1 for the as-treated population.

**Table 1:** Baseline characteristics according to treatment arm and regimen for the as-treated population

		Overall (*n* = 2182)	Standard arm (*n* = 1095)			Experimental arm (*n* = 1087)
			NF_seq_ (*n* = 120)	NF_SIB_ (*n* = 605)	HF_seq_ (*n* = 370)	HF_SIB_ (*n* = 1087)
Age	<50 years	436 (20.0%)	29 (24.2%)	110 (18.2%)	73 (19.7%)	224 (20.6%)
	50–69 years	1487 (68.1%)	85 (70.8%)	418 (69.1%)	254 (68.6%)	730 (67.2%)
	≥70 years	259 (11.9%)	6 (5.0%)	77 (12.7%)	43 (11.6%)	133 (12.2%)
pT	T0	151 (6.9%)	9 (7.5%)	38 (6.3%)	24 (6.5%)	80 (7.4%)
	T1a	136 (6.2%)	5 (4.2%)	47 (7.8%)	23 (6.2)	61 (5.6%)
	T1b	452 (20.7%)	28 (23.3%)	136 (22.5%)	63 (17.0%)	225 (20.7%)
	T1c	956 (43.8%)	49 (40.8%)	255 (42.1%)	182 (49.2%)	470 (43.2%)
	T1mi	11 (0.5%)	0 (0%)	2 (0.3%)	4 (1.1%)	5 (0.5%)
	T2	441 (20.2%)	27 (22.5%)	119 (19.7%)	71 (19.2%)	224 (20.6%)
	T3	11 (0.5%)	1 (0.8%)	3 (0.5%)	3 (0.8%)	4 (0.4%)
	T4b	4 (0.2%)	0 (0%)	1 (0.2%)	0 (0%)	3 (0.3%)
	Tis	19 (0.9%)	1 (0.8%)	4 (0.7%)	0 (0%)	14 (1.3%)
	Unknown	1 (0%)	0 (0%)	0 (0%)	0 (0%)	1 (0.1%)
pN	N0	2044 (93.7%)	108 (90.0%)	574 (94.9%)	344 (93.0%)	1018 (93.7%)
	N1a	80 (3.7%)	9 (7.5%)	17 (2.8%)	11 (3%)	43 (4%)
	N1mi	50 (2.3%)	3 (2.5%)	12 (2.0%)	14 (3.8%)	21 (1.9%)
	Unknown	8 (0.4%)	0 (0%)	2 (0.3%)	1 (0.3%)	5 (0.5%)
cM	M0	2099 (96.2%)	118 (98.3%)	582 (96.2%)	351 (94.9%)	1048 (96.4%)
	Unknown	83 (3.8%)	2 (1.7%)	23 (3.8%)	19 (5.1%)	39 (3.6%)
Grading	G1 well differentiated	512 (23.5%)	27 (22.5%)	156 (25.8%)	77 (20.8%)	252 (23.2%)
	G2 moderately differentiated	1151 (52.7%)	65 (54.2%)	329 (54.4%)	191 (51.6%)	566 (52.1%)
	G3 poorly differentiated	499 (22.9%)	28 (23.3%)	117 (19.3%)	96 (25.9%)	258 (23.7%)
	Unknown	20 (0.9%)	0 (0%)	3 (0.5%)	6 (1.6%)	11 (1.0%)
ECOG	0	1629 (74.7%)	81 (67.5%)	470 (77.7%)	268 (72.6%)	810 (74.6%)
	1	540 (24.8%)	35 (29.2%)	135 (22.3%)	101 (27.4%)	269 (24.8%)
	2	10 (0.5%)	4 (3.3%)	0 (0%)	0 (0%)	6 (0.6%)
	Unknown	1 (0%)	0 (0%)	0 (0%)	0 (0%)	0 (0%)
Chemotherapy status	None	1431 (65.6%)	74 (61.7%)	427 (70.6%)	238 (64.3%)	692 (63.7%)
	Preoperative	406 (18.6%)	23 (19.2%)	87 (14.4%)	85 (23.0%)	211 (19.4%)
	Postoperative	342 (15.7%)	23 (19.2%)	91 (15.0%)	47 (12.7%)	181 (16.7%)
	Unknown	2 (0.1%)	0 (0%)	0 (0%)	0 (0%)	2 (0.2%)
Chemotherapy	FEC	8 (1.1%)	2 (4.3%)	3 (1.7%)	1 (0.8%)	2 (0.5%)
	FEC-Doc	14 (1.9%)	1 (2.2%)	6 (3.5%)	4 (3.1%)	3 (0.8%)
	TC	22 (3.0%)	2 (4.3%)	4 (2.3%)	4 (3.1%)	12 (3.2%)
	Others	543 (75.1%)	29 (63.0%)	132 (76.7%)	86 (66.7%)	296 (78.7%)
	Unknown	6 (0.8%)	0 (0%)	3 (1.7%)	1 (0.8%)	2 (0.5%)

Data presented as number of patients, with percentages in parentheses

*NF*_*seq*_ conventionally fractionated radiotherapy with sequential boost, *NF*_*SIB*_ conventionally fractionated radiotherapy with simultaneous boost, *HF*_*seq*_ hypofractionated radiotherapy with sequential boost, *ECOG* Eastern Cooperative Oncology Group, *FEC* 5-fluorouracil epirubicin cyclophosphamide, *Doc* docetaxel, *TC* docetaxel-cyclophosphamide

### Fractionation patterns in the standard arm

In the standard arm, 605 patients (55.3%) received NF_SIB_, 370 patients had HF_seq_ (33.8%), and 120 patients (11%) were treated with NF_seq_ (Table 2). 830 and 265 patients were enrolled prior to and after the S3 guideline update, respectively. For NF_seq_, there was no difference between 11.33% and 9.81% of patients prior to and after the guideline publication, respectively (RR = 0.96, 95% CI = [0.88; 1.08]). There was a decrease in the use of NF_SIB_ from 59.04 to 43.40% (RR = 0.86, 95% CI = [0.80; 0.92]), and an increase in the use of HF_seq_ from 29.64 to 46.79% of patients (RR = 1.21, 95% CI = [1.12; 1.32]). For RRs per year and corresponding 95% CIs, see Table 2. Fig. 1 shows the temporal trend in fractionation patterns in the standard arm across quarter years of enrollment. When analyzing annual trends in the choice of fractionation in the standard arm, both a decrease in the use of NF_seq_ and an increase in the use of HF_seq_ were shown (Table 2). For non-academic institutions, NF_SIB_ was the most commonly applied fractionation regimen for every single quarter year of enrollment, while the use of HF_seq_ continuously increased during the enrollment period, approaching about 40% after the guideline update (Fig. 2). At university hospitals, the use of HF_seq_ reached 40% even before the guideline update and increased to about 80% afterwards.

**Table 2:** Frequency of different fractionation regimens in the standard treatment arm by time in breast cancer patients (as treated)

Fractionation regimen	Total	Before S3 guideline update	After S3 guideline update	RR (95% CI) [/year (95% CI)]
Conventional fractionation with sequential boost (NF_seq_)	120 (10.96%)	94 (11.33%)	26 (9.81%)	0.96 (0.88; 1.08) [0.91 (0.77; 1.07)]
Conventional fractionation with simultaneous integrated boost (NF_SIB_)	605 (55.25%)	490 (59.04%)	115 (43.40%)	0.86 (0.80; 0.92) [0.90 (0.86; 0.95)]
Hypofractionation with sequential boost (HF_seq_)	370 (33.79%)	246 (29.64%)	124 (46.79%)	1.21 (1.12; 1.32) [1.23 (1.14; 1.33)]

Data presented as number of patients, with percentages in parentheses. Relative risk (RR) per year estimated by generalized linear model assuming binomial distribution of patients in the respective category and identical link function

*RR* relative risk, *CI* confidence interval

Results of the multiple logistic regression models are shown in Table 3. The interaction between time and setting was significant for the scenario HF_seq_ vs. NF_seq_ and NF_SIB_ (nominal *p*-value = 4.18 × 10^−5^, adjusted *p*-value = 6.27 × 10^−5^) and for the scenario NF_SIB_ vs. HF_seq_ and NF_seq_ (nominal *p*-value = 1.19 × 10^−7^, adjusted *p*-value = 3.57 × 10^−7^). However, patients recruited at university hospitals were significantly more likely to be treated with HF_seq_ than patients enrolled at other institutions both before (OR = 1.7, 95% CI = [1.17; 2.33]) and after (OR = 6.4, 95% CI = [3.70; 11.14]) the guideline update.

**Table 3:** Multiple logistic regression models regarding treatment regimens in the standard treatment arm with interaction on time (before/after S3 guideline update) and setting (university hospitals vs. other institutions)

Variable	NF_seq_ vs. NF_SIB_ and HF_seq_			NF_SIB_ vs. NF_seq_ and HF_seq_			HF_seq_ vs. NF_seq_ and NF_SIB_		
	β	SE	*p*-value (*p*-adj)	β	SE	*p*-value (*p*-adj)	β	SE	*p*-value (*p*-adj)
Time	−0.74	0.37	0.0453	0.14	0.19	0.4685	0.12	0.20	0.5431
Age (years)	−0.02	0.01	0.0167	0.004	0.01	0.5467	0.01	0.01	0.3051
Chemotherapy	0.26	0.21	0.2045	−0.31	0.14	0.0250	0.21	0.14	0.1415
Tumor size (cm)	0.05	0.10	0.6439	−0.01	0.07	0.9121	−0.02	0.07	0.8299
Setting	0.44	0.24	0.0733	−0.65	0.17	1.00 × 10^−4^	0.50	0.18	0.0044
Time * setting	0.77	0.50	0.1221 (0.1221)	−2.11	0.40	1.19 × 10^−7^ (3.57 × 10^−7^)	1.36	0.33	4.18 × 10^−5^ (6.27 × 10^−5^)

Main effects time and setting are adjusted for age (in years), tumor size (in centimeter), and use of chemotherapy (yes/no)

*NF*_*seq*_ conventionally fractionated radiotherapy with sequential boost, *NF*_*SIB*_ conventionally fractionated radiotherapy with simultaneous boost, *HF*_*seq*_ hypofractionated radiotherapy with sequential boost, *β* effect estimate, *SE* standard error, *p‑value* descriptive *p*-value, *p‑adj.* adjusted *p*-values according to Bonferroni–Holm for interaction term

### Enrollment of patients with nodal involvement

A total of 93.9% of patients had no nodal involvement while 6.1% showed 1–2 involved lymph nodes. The distribution of nodal involvement per enrollment quarter year is shown in Fig. 3. The frequency of nodal involvement before the guideline update was 6.1% vs. 6.0% thereafter. There was no time trend for nodal involvement when analyzed per year and using the guideline update as cutoff date (see Table 4).

**Table 4:** Inclusion changes over time according to number of involved lymph nodes for 2170 patients with known lymph node status

Involved lymph nodes	Total	Before S3 guideline update	After S3 guideline update	RR (95% CI) [/year (95% CI)]
0 positive lymph nodes	2038 (93.92%)	1537 (93.89%)	501 (94.0%)	1.005 (0.897; 1.096) [0.979 (0.832; 1.153)]
1–2 positive lymph nodes	132 (6.08%)	100 (6.11%)	32 (6.0%)	1.015 (0.924; 1.151) [0.999 (0.990; 1.008)]

Data presented as number of patients, with percentages in parentheses. Relative risk (RR) per year estimated by generalized linear model assuming binomial distribution of patients in the respective category and identical link function

*RR* relative risk, *CI* confidence interval

## Discussion

Our analysis demonstrates an increased use of hypofractionated whole-breast radiotherapy in patients randomized to the standard arm of the HYPOSIB trial after publication of the updated S3 guideline as well as over the course of the entire trial. However, there was a significant interaction with treatment setting. Patients receiving treatment at university hospitals were more likely to receive hypofractionated radiotherapy. Only a minority of the enrolled patients had lymph node involvement, and no significant change in the proportion of lymph node-positive patients was detected during the enrollment period or after the guideline update.

Despite the encouraging long-term results of hypofractionated whole-breast radiotherapy, adoption in clinical practice has been slow, as shown by several population-based analysis from the United States and Australia [15–18]. Adoption of hypofractionated radiotherapy increased over time in all of the datasets. However, the degree of adoption depended on the studied timeframe and patient characteristics. Two clinical publications and one recent European survey that studied the impact of treatment setting (academic/hospital-associated vs. non-academic/free-standing practice) confirm our finding that adoption is more pronounced at academic facilities [16, 17, 19]. Interestingly, there was no visible impact of chemotherapy use on the choice of HF_seq_, despite other data suggesting that the adoption of hypofractionation is slower in this subgroup [16].

There are only limited data on adoption of hypofractioned radiotherapy for breast cancer in Germany. However, our findings are confirmed by a recent analysis of the German INSEMA trial, which studies de-escalation of axillary surgery in early-stage breast cancer. Despite enrolling mostly patients with low-risk characteristics, only 15.8% of patients received hypofractionated radiotherapy after breast-conserving surgery [20]. A recent survey publication conducted in 2017 in Germany revealed that there were significant reservations regarding hypofractionated radiotherapy for breast cancer [21]. Major points of concern voiced by participants were increased side effects, an impaired toxicity profile, and insufficient data, which stands in stark contrast to the published literature [22]. Lower reimbursement rates may further hamper implementation of hypofractionated radiotherapy for breast cancer, as stated by 19.9% of participants in the mentioned survey [21] as well as by 9.2% of participants in a recent European survey [19]. It is interesting to note that in our analysis, NF_SIB_ was the most commonly used fractionation regimen in the standard arm despite the relatively low quality of evidence from mostly dosimetric and cohort studies [23–29].

The 10-year results from the Ontario and the START A/B trials have been available since 2010 and 2013, respectively. The 2012 version of the interdisciplinary S3 guidelines advocated the optional use of hypofractionated radiotherapy for patients with low-risk features [7]. Apart from the German interdisciplinary S3 guidelines there are national guidelines from the *Arbeitsgemeinschaft Gynäkologische Onkologie* [30, 31], which are updated annually, as well as international guidelines, e.g., from the American Society for Radiation Oncology [32], which endorsed the preferential use of hypofractionated over normofractionated whole-breast radiotherapy in early 2017 and mid-2018. Thus, the increased use of HF_seq_ might also reflect a gradual implementation independent of the publication of the S3 guideline update.

The overall number of patients with lymph node involvement in the HYPOSIB trial was surprisingly low. At the time of trial conception, RNI was not recommended for patients with 1–3 involved lymph nodes. However, publication of several prospective trials of RNI in patients with limited nodal involvement during the early stages of the HYPOSIB trial showed improved outcomes [9, 10, 33]. Nevertheless, this was only implemented in the German S3 guidelines in 2017 [12]. Since conventional fractionation is regarded as the standard of care for patients with RNI [12, 30, 34], we hypothesized that a potential increase in the use of RNI might have impacted on the recruitment of patients with lymph node involvement for the HYPOSIB trial. There are several possible explanations for the low number of patients with lymph node involvement enrolled in the HYPOSIB trial. The results of the mentioned trials might have led to an early adoption of RNI even before implementation in national guidelines. In the era of decreasing radicality of axillary surgery following publication of the ACOSOG Z0011 trial in 2011 [35], clinicians might have felt less comfortable including patients with nodal involvement and a relevant risk of subclinical nodal disease into a trial of hypofractionated radiotherapy [36, 37]. Furthermore, other changes in treatment patterns, such as the increasing use of neoadjuvant chemotherapy [7] and competing clinical trials, might have had an influence on the enrollment of node-positive patients.

The main limitation of our retrospective analysis is that our findings may not apply to clinical reality outside of clinical trials. However, participation was broad, with 87 recruiting institutions including tertiary academic centers, non-academic hospitals, and private practices. The reasoning for choosing a specific fractionation regimen in the standard arm of the HYPOSIB trial was not documented. Since age, tumor size, tumor biology, and use of chemotherapy are not independent from each other, the ability to unequivocally discern the relative impact of these variables on fractionation choice is limited in our analysis.

## Conclusion

There was an increase in the use of hypofractionated radiotherapy in the standard arm over the course of the HYPOSIB trial and after publication of the S3 guideline update. This was primarily driven by patients enrolled at university hospitals. Enrollment of patients with lymph node involvement was low throughout the trial. Further analyses of the HYPOSIB trial are ongoing.
